# Minimal Yet Powerful: The Role of Archaeal Small Heat Shock Proteins in Maintaining Protein Homeostasis

**DOI:** 10.3389/fmolb.2022.832160

**Published:** 2022-05-12

**Authors:** Mousam Roy, Koustav Bhakta, Abhrajyoti Ghosh

**Affiliations:** Department of Biochemistry, Bose Institute, Kolkata, India

**Keywords:** small heat shock protein (sHsp), holdase, oligomerization, protein folding, aggregation protection

## Abstract

Small heat shock proteins (sHsp) are a ubiquitous group of ATP-independent chaperones found in all three domains of life. Although sHsps in bacteria and eukaryotes have been studied extensively, little information was available on their archaeal homologs until recently. Interestingly, archaeal heat shock machinery is strikingly simplified, offering a minimal repertoire of heat shock proteins to mitigate heat stress. sHsps play a crucial role in preventing protein aggregation and holding unfolded protein substrates in a folding-competent form. Besides protein aggregation protection, archaeal sHsps have been shown recently to stabilize membranes and contribute to transferring captured substrate proteins to chaperonin for refolding. Furthermore, recent studies on archaeal sHsps have shown that environment-induced oligomeric plasticity plays a crucial role in maintaining their functional form. Despite being prokaryotes, the archaeal heat shock protein repository shares several features with its highly sophisticated eukaryotic counterpart. The minimal nature of the archaeal heat shock protein repository offers ample scope to explore the function and regulation of heat shock protein(s) to shed light on their evolution. Moreover, similar structural dynamics of archaeal and human sHsps have made the former an excellent system to study different chaperonopathies since archaeal sHsps are more stable under *in vitro* experiments.

## Introduction

Sudden changes in environmental conditions can damage macromolecules inside a cell, putting the organism under stress. One of the major challenges under stress is to maintain protein homeostasis. Stress can cause denaturation and aggregation of proteins, which could lead to proteotoxicity. Proteotoxic stress could be detrimental for the cell if not taken care of (Macario et al., 1999). Chaperones are a ubiquitous group of proteins that help to maintain protein homeostasis under stress (Hall et al., 1995; Macario et al., 1999). Chaperone proteins are also known as heat shock proteins (Hsps) as they were first discovered in Drosophila salivary cells under heat stress (Ritossa, 1962). There are five major classes of heat shock proteins based on their molecular weight, namely Hsp100, Hsp90, Hsp70, Hsp60, and small heat shock proteins (Trent, 1996; Richter et al., 2010). Small heat shock proteins are the only ATP-independent chaperones that initiate an organism’s stress response (Haslbeck and Vierling, 2015; Haslbeck et al., 2019). Small heat shock proteins (sHsps) have been extensively studied in bacteria and eukaryotes (Van Montfort et al., 2001; Baranova et al., 2011; Haslbeck and Vierling, 2015; Zwirowski et al., 2017; Haslbeck et al., 2019). However, in the third domain of life, archaea, the study of sHsps was considerably less until recently. This is surprising given the added importance of sHsps in archaea due to their limited repertoire of Hsps. Most archaea do not possess Hsp100 and Hsp90 (Macario et al., 1999; Robinson et al., 2018). Moreover, in thermophilic and hyperthermophilic archaea, Hsp70 remains absent, which is considered a central player of the stress response pathway in bacteria and eukaryotes (Macario et al., 1999; Usui et al., 2004; Robinson et al., 2018). Another interesting fact about archaea is that many of their heat shock proteins are closely related to eukaryotes rather than bacteria. For example, many archaea possess group II chaperonin, a homolog of which is also present in the eukaryotic cytosol (Macario et al., 1999; Chaston et al., 2016). Therefore, a detailed analysis of archaeal heat shock proteins might provide valuable information about their eukaryotic counterpart. Furthermore, establishing archaea as a model system to study and understand the molecular mechanics of their function could help our understanding of proteotoxic diseases like Alzheimer’s or Perkinson. Here, in this review, we discuss the recent advances in archaeal small heat shock proteins and stress response to get a better idea about the system and understand the future questions that need to be addressed.

## Oligomeric Plasticity and sHsp Function

**FIGURE 1:**
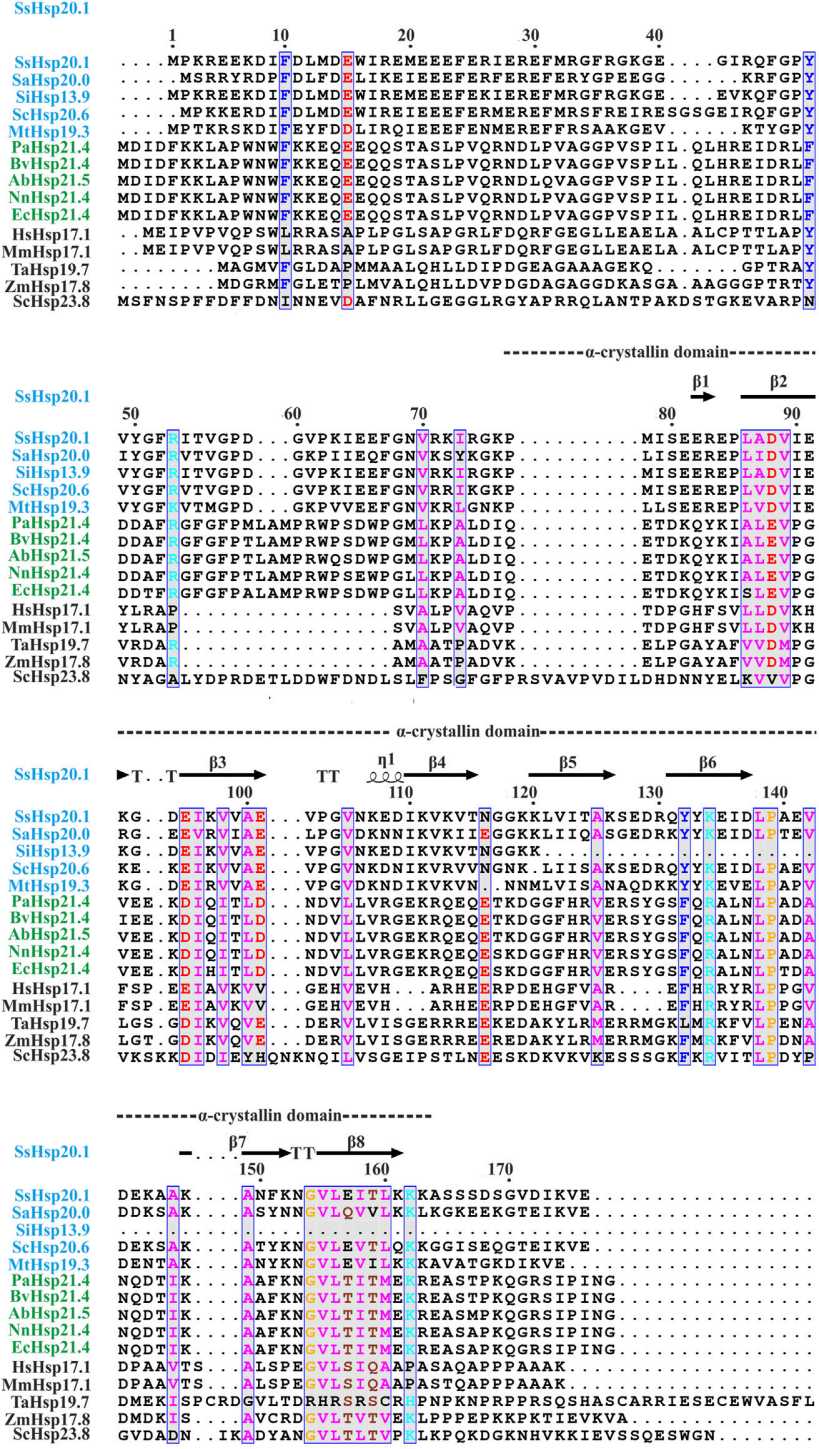
Amino acid sequence alignment of Hsp20 protein family from archaea (blue), bacteria (green), and eukaryotes (black). Secondary structural domains at the top are based on SsHsp20.1 ACD (4-RZK) crystal structure. Amino acids in blue boxes represent similarity across groups. Abbreviations for sHsps: SsHsp20.1 (*Sulfolobus solfataricus* Hsp20.1), SaHsp20.0 (*Sulfolobus acidocaldarius* Hsp20.0), SiHsp13.9 (*Sulfolobus islandicus* Hsp13.9), ScHsp20.6 (*Saccharolobus caldissimus* Hsp20.6), MtHsp19.3 (*Metallosphaera tengchongensis* Hsp19.3), PaHsp21.4 (*Pseudomonas aeruginosa* Hsp21.4), BvHsp21.4 (*Burkholderia vietnamiensis* Hsp21.4), AbHsp21.5 (*Acinetobacter baumannii* Hsp21.5), NnHsp21.4 (*Nitrosomonas nitrosa* Hsp21.4), EcHsp21.4 (*Escherichia coli* Hsp21.4), HsHsp17.1 (*Homo sapiens* Hsp17.1), MmHsp17.1 (*Macaca mulatta* Hsp17.1), TaHsp19.7 (*Triticum aestivum* Hsp19.7), ZmHsp17.8 (*Zea mays* Hsp17.8), ScHsp23.8 (*Saccharomyces cerevisiae* Hsp23.8). Multiple sequence alignment was carried out using ESPript 3.0 and Clustal W.

**FIGURE 2:**
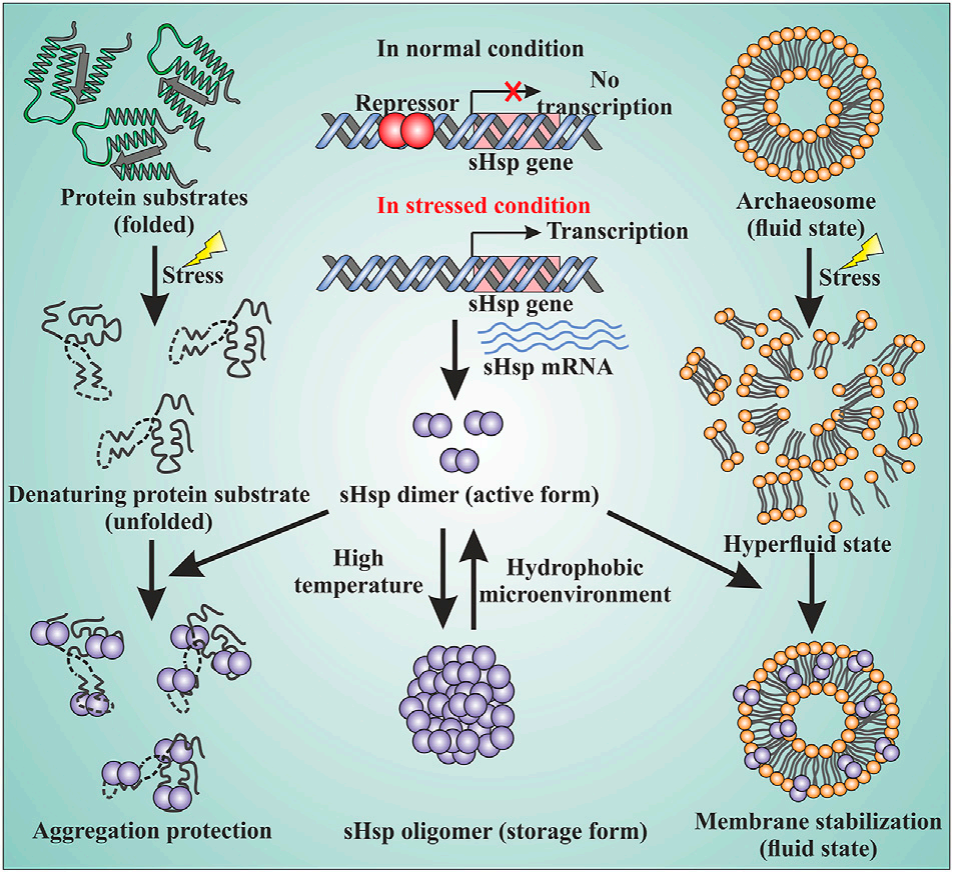
sHsps in archaea are involved in various cellular functions. The sHsp genes are under the control of transcriptional regulators. Under normal growth condition, a repressor binds to the upstream of the genes and the expression of the genes are turned off. Under the stressed condition, the repressor can no longer bind to DNA and the sHsp genes are expressed. The dimer is the active form of sHsps. At high temperatures, the dimers can associate with each other and form a large oligomeric structure which is the storage form of the sHsps (middle panel). Under the stressed condition, the native proteins of the cell start to unfold. The unfolded proteins can lead to the formation of large amorphous aggregates. sHsps bind to the unfolded proteins by their hydrophobic patches and protect against stressed-induced aggregation (left panel). Stress can also lead to membrane destabilization. sHsps can bind to the membrane and thereby maintain membrane fluidity (right panel) by stabilizing the membrane.

Small heat shock proteins are usually composed of three structural elements, a highly variable and flexible N terminal domain flanked by a β-sheet rich α-crystallin domain and a C-terminal domain containing charge residues (Laksanalamai and Robb, 2004; Haslbeck and Vierling, 2015) (Figure 1). Most small heat shock proteins form dimers that associate with each other to form large polydisperse oligomeric structures (Kim K. K. et al., 1998; Kim et al., 1998b; Ruepp et al., 2001). The polydispersity of oligomeric structures is crucial for the function, as demonstrated in Ta16.9 and Ps18.1 (Santhanagopalan et al., 2018). In both these sHsps, introduction of disulfide bonds in non-dimeric interfaces prevent their ability to dissociate into dimeric forms, resulting in a reduction of their activity (Santhanagopalan et al., 2018). Besides activation of sHsps, polydispersity can also help to recognize different substrates by the same sHsp. For example, human HspB1 forms different oligomeric structures that can interact with different substrates (Mani et al., 2016). An I-X-I motif in the C-terminal region plays a crucial role in forming the oligomer (Saji et al., 2008; Delbecq et al., 2012; Liu et al., 2015). These highly polydisperse oligomeric forms of sHsp are dynamic and can dissociate into small dimeric forms depending on the environment (Stengel et al., 2010; Basha et al., 2012; Nandi et al., 2015; Roy et al., 2018). Hsp16.9 and Hsp18.1 from *Triticum aestivum* (Ta16.9) and *Pisum sativum* (Ps18.1), respectively, dissociate into an active dimeric form from a dodecameric oligomeric structure (Santhanagopalan et al., 2018). On the other hand, evidence of oligomer being the active form is also present. In *Caenorhabditis elegans*, upon increasing the temperature, Hsp17 transforms from a spherical oligomer to a large sheet-like supermolecular assembly (SMA) which is the functional form (Zhang et al., 2015). In the archaeal domain of life, recent studies have established the dimer as the active form of sHsp (Liu et al., 2015; Roy et al., 2018). In thermoacidophilic archaeon, *Sulfolobus acidocaldarius* Hsp20 forms a large 24-mer structure at room temperature that dissociates into dimeric forms upon increasing the hydrophobicity of the solution (Figure 2) (Roy et al., 2018). Hsp20.1 from another thermoacidophilec archaeon, *Sulfolobus solfataricus* showed that dimer can protect the substrate from heat-induced aggregation (Liu et al., 2015). In *Sulfolobus acidocaldarius*, high temperatures lead to the formation of large oligomeric storage ensembles for both Hsp20 and Hsp14 (Figure 2) (Roy et al., 2018). Together, all these studies show that sHsps are highly polydisperse and undergo conformational changes in their oligomeric structures resulting in the formation of an active functional form. A detailed view of representative sHsps in all three domains of life and the sequence alignment depicting structural and functional domains are presented in Figure 1.

## Different Functions of Small Heat Shock Proteins

**FIGURE 3:**
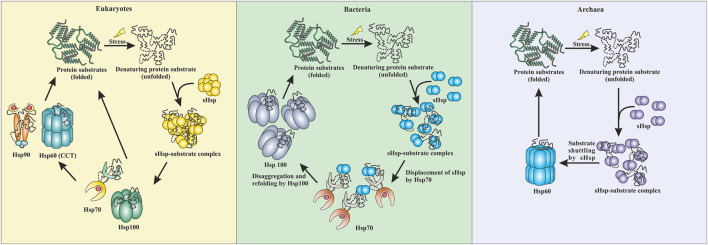
Comparative representation of stress response pathway in three domains of life. In eukaryotes, sHsps bind to the unfolded proteins and prevent aggregation. The unfolded proteins can then be transferred to the Hsp70 pr Hsp100 system. They can refold a protein back to their native conformations. Additionally, Hsp90 can break the deadlock created by Hsp70 and can perform the refolding process. CCT (Hsp60) can also refold a protein back to its native conformation. In bacteria, the sHsps protect against aggregation by binding to unfolded proteins when subjected to stress. Hsp70 displaces the sHsps and takes over the refolding process. Additionally, Hsp100 (disaggregases) comes into the scenario and gets the final refolding complete. Similarly, in archaea, sHsps are the first line of defense. They protect the cell against stress-induced aggregation by binding to unfolded proteins. The unfolded protein substrates are then transferred by sHsps to Hsp60 (thermosome), which performs the final refolding.

Small heat shock proteins are the first line of defense against proteotoxicity in an organism (Figure 3) (Haslbeck and Vierling, 2015; Haslbeck et al., 2019). The unfolding of proteins opens up their buried hydrophobic patches, which interact to form protein aggregates. The unfolding of cytosolic proteins also increases hydrophobicity inside the cell. Small heat shock proteins respond to this increased hydrophobicity by dissociating into dimeric forms as mentioned in the previous section. The dimeric form of sHsps also undergoes conformational changes that increase the number of surface-exposed hydrophobic patches. These hydrophobic patches of sHsps interact with the hydrophobic patches of an unfolded protein and prevent self-interaction and aggregation of the unfolded proteins. This is known as the “holdase” function of sHsps (Haslbeck et al., 2005; Haslbeck and Vierling, 2015; Ungelenk et al., 2016). Small heat shock proteins, however, can not refold the unfolded protein due to the lack of ATPase function. Hsp70 is then recruited into the complex for further processing of the unfolded substrate. (Lee and Vierling, 2000; Mogk et al., 2003; Zwirowski et al., 2017). The “holdase” function is ubiquitous for sHsps across all three domains of life (Figure 3). Diverse functions of sHsp are also reported in bacteria and eukaryotes (Table 1; Figure 3). For example, *E. coli* Hsp15 protects DNA by non-specifically getting associated with it (Korber et al., 1999; Macario et al., 1999). In yeast, Hsp42 initiates aggregation of the unfolded proteins and forces them to precipitate, thus preventing them from acting as nucleation points for the aggregation of native proteins (Ungelenk et al., 2016). In archaea, sHsps prevent protein aggregation through their “holdase” function. This generalized mechanism of detection of hydrophobicity and prevention of unfolded substrate proteins give robustness in the heat shock response repositoire especially in archaea where only a limited number of sHsps is present. Several reports on other functions of sHsps are also evident among the archaeal representatives (Table 1). For example, in *Sulfolobus acidocaldarius* Hsp20 is secreted in the extracellular vesicles and prevents membrane destabilization under heat stress (Figure 2) (Roy et al., 2018). It has been demonstrated that the destabilization of the membrane also increases the hydrophobicity of the solution like the unfolded proteins (Roy et al., 2018). Hsp20 responds to this increased hydrophobicity and dissociates into the dimeric form opening up its hydrophobic patches. The dimeric form of Hsp20 then interacts with the membrane and prevents further destabilization (Roy et al., 2018). In *Sulfolobus tokodaii*, Hsp20 is reportedly overexpressed during biofilm formation (Koerdt et al., 2011). In the absence of Hsp70, archaeal sHsps can also transfer associated substrate to Hsp60 for refolding.

**TABLE 1:** A survey of sHsp compositions, molecular weight, and function from all three domains of life. *Homo sapiens*, *Escherichia coli* k12, and *Sulfolobus acidocaldarius* have been presented as representatives from eukaryotes, bacteria, and archaea.

Domain of life	Organism	Protein	Gene	Molecular weight (kDa)	Known molecular function	References
Eukaryote	Human (*Homo sapiens*)	HSPB1	*hspb1*	22.7	1. Acts as a molecular chaperone maintaining denatured proteins in a folding-competent state	Rogalla et al. (1999), Kostenko et al. (2009). Almeida-Souza et al. (2010), Holmgren et al. (2013)
					2. Plays a role in stress resistance and actin organization	
					3. Axonal transport of neurofilament proteins	
		HSPB2	*hspb2*	20.2	1. Regulates the kinase DMPK.	Suzuki et al. (1998)
		HSPB3	*hspb3*	16.9	1. Acts as a molecular chaperone preventing heat-induced aggregation	Asthana et al. (2012)
		HSPB4	*cryaa*	19.9	1. Contributes to the transparency and refractive index of the lens	Murugesan et al. (2007), Richter et al. (2008), Bhagyalaxmi et al. (2009), Nagaraj et al. (2012), Kaiser et al. (2019)
					2. Oxidized form acts as a chaperone	
					3. Corrects the formation of lens intermediate filaments	
		HSPB5	*cryab*	20.1	1. Exhibits chaperone-like activity, preventing aggregation of various proteins	Delbecq and Klevit, (2019)
		HSPB6	*hspb6*	17.1	1. Acts as a molecular chaperone	Bukach et al. (2004)
		HSPB7	*hspb7*	18.6	1. Suppresses polyQ aggregation and prevents polyQ-induced toxicity in cells	Vos et al. (2010)
		HSPB8	*hspb8*	21.6	1. Acts as a molecular chaperone and prevents polyQ aggregation	Carra et al. (2008)
		HSPB9	*hspb9*	17.4	1. Expressed in testes and plays some important sex-related role	Kappé et al. (2001)
		HSPB10	*odf1*	28.3	1. Component of the outer dense fibers (ODF) of spermatozoa	Liu et al. (2011)
					2. Helps to maintain the passive elastic structures and elastic recoil of the sperm tail	
Bacteria	*Escherichia coli* k12	IbpA	*ibpa*	15.7	1. Prevents the aggregation of denatured proteins	Zwirowski et al. (2017)
		IbpB	*ibpb*	16.0	1. Prevents the aggregation of denatured proteins	Zwirowski et al. (2017)
Archaea	*Sulfolobus acidocaldarius*	Hsp14	*saci_1665*	14.3	1. Protects unfolded protein from aggregation	Roy et al. (2021)
					2. Delivers heat inactivated substrate to group II chaperonin	
		Hsp20	*saci_0922*	19.9	1. Protects unfolded protein from aggregation	Roy et al. (2018)

This phenomenon has been observed in *Mycobacterium butanii*, *Pyrococuus furiosus,* and also in *Sulfolobus acidocaldarius* (Laksanalamai et al., 2006; Laksanalamai et al., 2009; Luo et al., 2009; Roy et al., 2021). It has been observed that excess addition of sHsp from *Thermococcus sp*. KS-1 can also refold back chemically denatured green fluorescence protein in an ATP-independent manner (Laksanalamai and Robb, 2004).

## Crosstalk Between Heat Shock Proteins and Substrate Transfer

Interactions between different heat shock proteins play a vital role for an organism to thrive in harsh conditions. Such communications are well documented in both eukaryotes and prokaryotes (Lee and Vierling, 2000; Mogk et al., 2003; Ungelenk et al., 2016; Zwirowski et al., 2017). Hsp70 remains the central player in all these interactions. Hsp70 interacts with the sHsp-substrate complex and disaggregates the sHsp associated unfolded proteins with the help of Hsp100 (Lee and Vierling, 2000; Mogk et al., 2003; Mogk et al., 2015; Zwirowski et al., 2017). The disaggregated substrate then gets refolded by Hsp70 itself or other ATP-dependent chaperones like Hsp90 or Hsp60 (Mogk et al., 2003; Kabir et al., 2011; Zwirowski et al., 2017; Moran Luengo et al., 2018). Many of these interactions are absent in archaea due to the limited repertoire of high molecular weight chaperones like Hsp100 or Hsp90 (Holmes et al., 2014; Lemmens et al., 2018). Furthermore, in hyperthermophilic and some thermophilic archaea, Hsp70 is also absent (Usui et al., 2004). Interestingly, it has recently been demonstrated that in the absence of Hsp70, sHsps of these organisms could directly transfer the associated substrate protein to Hsp60 for refolding (Laksanalamai et al., 2006; Laksanalamai et al., 2009; Roy et al., 2021). Studies have shown that *Pyrococcus furiosus* Hsp60 refolds heat-inactivated Taq polymerase five-fold more efficiently in the presence of sHsp (Laksanalamai et al., 2006). In *Sulfolobus acidocaldarius*, Hsp14 can bind to the unfolded substrate and is also involved in shuttling the unfolded substrate to Hsp60 for final ATP-dependent refolding (Figure 3) (Roy et al., 2021). Other than thermophilic archaea, transfer of the unfolded substrate from sHsp to Hsp60 was also reported in *Methanococcoides burtonii*, a cold adaptive archaeon (Laksanalamai et al., 2009). However, the exact mechanism of substrate shuttling between sHsps and Hsp60 is not yet clear. Recently, a direct physical interaction between Hsp14 and Hsp60 is reported in *Sulfolobus acidocaldarius* (Roy et al., 2021). In bacteria, a substrate transfer between Hsp60 and sHsp was also demonstrated in *Deinococcus radiodurans*, however, unlike archaea, substrate transfer between sHsp and Hsp70 remains the predominant refolding pathway (Bepperling et al., 2012).

## Interaction Between Small Heat Shock Proteins

Hetero-oligomer formation by sHsp is common in eukaryotes and bacteria (Stengel et al., 2010; Mymrikov et al., 2012; Arrigo, 2013; Zwirowski et al., 2017; Mymrikov et al., 2020). In general, small heat shock proteins form dynamic oligomeric ensembles. Within the cellular milieu, they also form hetero-oligomers. However, only the presence of different sHsps does not necessarily ensure the formation of heterooligomeric ensembles. For example, yeast has two sHsp, namely Hsp26 and Hsp42, which do not interact with each other (Ungelenk et al., 2016). The possibility of hetero-oligomer formation depends on the sHsps and the organism. In humans, eye lens chaperone proteins αA crystallin and αB crystallin form hetero-oligomers that are shown to be essential for their function (Biswas and Das, 2004). Two cytosolic sHsps from *Pisum sativum* Hsp17.9 and Hsp18.1 are also reported to form hetero-oligomer (Stengel et al., 2010). In eukaryotes, hetero-oligomeric structures help regulate sHsp’s function and achieve substrate specificity (Haslbeck and Vierling, 2015; Mymrikov et al., 2020). In bacteria, hetero-oligomers formation is also evident. In *E. coli*, two sHsps, IbpA and IbpB, form a co-complex that gets associated with unfolded substrate proteins and recruits Hsp70 for downstream substrate processing (Zwirowski et al., 2017). In archaea, only single evidence of such hetero-oligomer formation is reported to date in *Sulfololobus acidocaldarius*, where Hsp20 and Hsp14 can dynamically associate with each other to form a co-complex (Roy et al., 2021). Moreover, the hetero-oligomer formation between Hsp14 and Hsp20 has been shown to occur at temperatures beyond 50°C when the subunit exchange rate crosses a certain threshold (Roy et al., 2021). Hetero-oligomer formation in *Sulfololobus acidocaldarius* is crucial, as one of these sHsps is responsible for substrate transfer to Hsp60 for final refolding.

## Regulation of Archaeal Small Heat Shock Protein Expression

The area of gene regulation in archaea is poorly understood. The basal transcriptional machinery of archaea is fundamentally related to that of eukaryotes consisting of a complex multi-subunit RNAP and two general transcription factors (GTF). These two GTFs, namely TBP and TFB, are homologs of eukaryotic TATA-box binding protein and transcription factor IIB (TFIIB), respectively. During the process of transcription, TBP first binds to TATA-box and results in the bending of DNA and promoter region. TFB then binds to the TBP-DNA complex in BRE (TFB recognition element) upstream of the TATA-box. These interactions recruit RNAP to the promoter region, thus initiating transcription (Geiduschek and Ouhammouch, 2005). However, despite the similarities of the archaeal transcriptional machinery with eukaryotes, many putative transcriptional regulators identified in archaea resemble bacteria (Vierke et al., 2003; Geiduschek and Ouhammouch, 2005; Lu et al., 2008). Heat shock response is a well-established and widespread cellular phenomenon that is found across all three domains of life. Although many heat shock proteins have been identified in the domain of archaea, homologs of eukaryotic proteins like heat shock factors (HSF) or heat shock elements (HSE) have not been identified in archaea (Lu et al., 2008). To date, only two proteins, Phr in *Pyrococcus furiosus* and HSR1 in *Archaeoglobus fulgidus*, involved in transcriptional regulation of heat shock response in archaea have been identified. These two proteins are distantly related which not only regulates small heat shock genes but also autoregulates their own expression. In hyperthermophilic archaeon *Pyrococcus furiosus* a transcriptional regulator of heat shock genes, Phr was reported. Phr binds to a 29 bp DNA sequence that overlaps with the transcription start site. Binding of Phr to DNA inhibits transcription of *hsp20* gene in *Pyrococcus furiosus*. Inhibition of transcription occurred because, upon binding of Phr, RNA pol cannot bind the TBP/TFB promoter complex. Three consensus sequences of heat shock promoters are required for Phr binding which is TTTA at −10, TGGTAA at transcription start site, and AAAA at +10. The rise in growth temperature of this organism from 95°C to 103°C resulted in a decrease in the protein level of Phr. The mechanism suggests that at normal conditions Phr inhibits transcription of heat shock responsive genes. Upon increase in temperature *i.e.*, during heat stress, Phr no longer inhibits transcription thereby enabling the production of heat shock responsive proteins (Vierke et al., 2003; Karr, 2014). Not much information regarding the transcriptional regulation mechanism of HSR1 is available. HSR1 is also an autoregulatory protein that binds to the *cis*-binding motif, present upstream of its own gene in the sequence CTAAC-N5-GTTAG. The gene of HSR1 is part of an operon where a sHsp gene (*hsp20*) is present in its downstream. From the information of DNA binding location, it has been suggested that HSR1 binds to DNA under normal growth conditions of *A. fulgidus* (*i.e*., 78°C) and blocks the access of transcription machinery, which leads to repression of transcription. At higher temperatures, HSR1 can no longer bind to DNA thereby allowing transcription to occur (Rohlin et al., 2005; Karr, 2014). However, such a mechanism of transcriptional regulation of heat shock genes has not been identified in halophilic archaea. In *Haloferax volcanii* and *Halobacterium salinum,* the sequence of the BRE element and TATA-box of the core promoter of a small heat shock protein Hsp5 (P_hsp5_) was sufficient for heat shock response. In these organisms, increased expression of both the general transcription factors (TFB2 and TFBb) during heat shock has been reported. Such overexpressed GTFs further bind the promoter sequence of the *hsp5* gene and increase the cellular expression of Hsp5 (Lu et al., 2008).

## Establishment of Archaea as a Model System

Small heat shock proteins are ubiquitous across all three domains of life, emphasizing their importance in maintaining protein homeostasis. In humans, there are 10 different classes of sHsps that are active in different tissues and organs (Webster et al., 2019). Malfunction in these sHsps can lead to several diseases, from cataracts to Alzheimer’s (Kourtis and Tavernarakis, 2018; De Macario et al., 2019). Therefore, understanding sHsps role in disease prevention is crucial for therapeutic progress against such diseases. However, working with human sHsps is often difficult due to their labile nature under *in vitro* conditions. Also, a mutation in these sHsps can completely disrupt their structures. The loss of structure can be lethal and leads to no phenotype (De Macario et al., 2019). Therefore, working in a system in which sHsps have a significantly higher tolerance level is preferable. Archaea have the potential to act as the preferred organism to study sHsps role in disease. Archaea are the natural inhabitants of some of the most extreme environments on earth. Therefore, sHsps from the resident archaeal organisms in these habitats are usually exceptionally stable, making them suitable for studying the effect of different mutations that will otherwise completely disrupt human sHsps. Such high stability also makes them easy to be examined under various stress conditions *in vitro*. Another crucial advantage of using this system is the presence of a minimum repertoire of heat shock proteins. In a complex eukaryotic system, redundancy of the function of small heat shock proteins is one of the major challenges for researchers. Using archaeal models, one can address functional intricacies with the tools of genetic manipulation. Finally, certain phylum in archaea like crenarchaeota is more closely related to eukaryotes than bacteria allowing extrapolation of the results obtained in these organisms in the context of human diseases (Williams et al., 2013). Despite having many benefits of establishing archaea as a model organism, there are still several hurdles that need to be overcome. For example, many archaeal systems lack genetic tools, which make *in vivo* studies difficult to conduct. Also, there may be certain mechanistic differences between eukaryotic and archaeal sHsps despite their similarity, which may lead to an erroneous interpretation. Therefore, a thorough study and careful interpretation are essential before coming to any conclusion from studies on archaeal sHsps.

## Conclusion

In summary, we have discussed that small heat shock proteins play a momentous role inside a cell to maintain protein homeostasis. Small heat shock proteins are ATP-independent molecular chaperones that are present across all domains of life. They possess a conserved α-crystallin domain flanked by a C-terminal and variable N-terminal region. They form dynamic oligomeric ensembles and in many of them dimeric form is the active form and there exists an oligomer dimer equilibrium. In archaea, an increase in temperature leads to the formation of large oligomers. Dimeric sHsp can protect against stress-induced aggregation by binding to unfolded protein substrates via their hydrophobic patch. Besides protecting against aggregation, sHsps also interact with the membrane and confers stability to the membrane (Figure 2). sHsps cannot refold a substrate to its native structure. The refolding is done by group II chaperonin (Hsp60) in archaea. It has been observed that in archaea sHsp can transfer the unfolded substrate protein to Hsp60 (Figure 3). Not only that but also sHsp can physically interact with Hsp60. The sHsps in archaea also interact with each other and form hetero-oligomeric structures. During stressed conditions, the expression of sHsps is upregulated several folds. Although not much is known about the transcriptional regulation of sHsps in archaea, there are few mentions of regulatory proteins. These regulatory proteins bind to DNA sequences under normal conditions and inhibit the transcription of sHsps genes. Under stressed conditions, the regulatory proteins can no longer remain bound to DNA thereby allowing RNA polymerase to transcribe the genes of sHsps (Figure 2). Finally, we have seen that archaea can be an excellent model system to study the functional mechanism, mutational effects, and roles of sHsps in neurodegenerative disease because of their inherent stability. Although the sHsps in archaea have been explored to some extent, more work needs to be carried out. The precise details of unfolded substrate recognition by archaeal sHsps remain an enigma. Moreover, the substrate specificity is yet to be defined. Studying the mechanistic details and interface of interaction between archaeal sHsps will provide a profound understanding of their role and mechanism of action *in-vivo*. The mechanics of substrate transfer from sHsp to chaperonin as well as the intricacies of physical interactions between sHsps and chaperonin are yet to be explored vastly. Besides, there remain numerous questions regarding the regulation of sHsps at the molecular level. Addressing these questions might provide a platform to utilize archaeal sHsps in answering many fundamental questions related to the role of sHsps in cellular stress physiology.
